# Intravascular Ultrasound-Guided Stenting of the Celiac Artery for Hepatic Hypoperfusion After Acute Type A Aortic Dissection: A Case Report

**DOI:** 10.7759/cureus.60566

**Published:** 2024-05-18

**Authors:** Hailey B Shoemaker, Aldin Malkoc, Amira Barmanwalla, Raja Gnanadev, Amanda Daoud, Michelle Lee, Majid Tayyarah

**Affiliations:** 1 Surgery, Kaiser Permanente Bernard J Tyson School of Medicine, Pasadena, USA; 2 General Surgery, Arrowhead Regional Medical Center, Colton, USA; 3 Vascular Surgery, Kaiser Permanente Fontana Medical Center, Fontana, USA

**Keywords:** ultrasound-guided stent placement, hepatic malperfusion, celiac artery dissection, type a aortic dissection, celiac artery stent

## Abstract

Type A aortic dissection is a life-threatening emergency requiring prompt surgical treatment. The dissection itself and use of cardiopulmonary bypass can lead to further postoperative complications, including aortic branch occlusion, thrombosis, ischemia, and fatal end-organ damage. Celiac artery occlusion with consequent hepatic malperfusion is one feared complication of aortic dissection, which requires urgent surgical intervention. Optimal management of celiac artery dissection in the setting of type A aortic dissection has not yet been described in the literature. In this report, we describe a 39-year-old female patient with hypertension who was found to have celiac artery dissection and impending hepatic failure less than 48 hours after emergent ascending aortic replacement for type A aortic dissection. Placement of an ultrasound-guided endovascular celiac artery stent enabled reperfusion of the liver, ultimately saving the patient’s life.

## Introduction

Aortic dissection is among the most common and fatal cardiovascular emergencies, with mortality rates ranging from 40 to 50% within 48 hours of onset [1,2]. This phenomenon occurs when damage to the aortic wall causes a tear in the intima and diverts the high-pressure aortic blood flow into a false lumen. Type A aortic dissection (TAAD) involves both the ascending and descending aorta and requires urgent surgical repair. TAAD repair procedures generally require the use of a cardiopulmonary bypass (CPB), during which perfusion to intraabdominal organs is significantly decreased [1,3]. Complications of TAAD and CPB include renal insufficiency, hepatic dysfunction, and/or bowel ischemia, which increase rates of postoperative mortality [4]. Tears in the aortic wall can extend into the main branches, including the celiac artery and superior mesenteric artery, which supply blood to the foregut and midgut, respectively. The presence of a mural thrombus or periarterial fat infiltration should raise suspicion for celiac artery dissection, which can be confirmed with the presence of an intimal flap [5,6].

Complications of celiac artery dissection include aneurysm, rupture, and end-organ damage, such as acalculous ischemic cholecystitis, pancreatitis, and hepatic dysfunction [4,7,8,9]. In the setting of mesenteric ischemia, resolution of the ischemia takes precedence over central aortic repair. However, the risk of bowel ischemia secondary to a celiac trunk dissection is lower than that of a superior mesenteric artery dissection [5,10]. Treatment options for celiac artery dissection include resection of the diseased segment with subsequent anastomosis, bypass, or stent placement, followed by long-term antiplatelet, anticoagulation, and antihypertensive therapy [4,5,11,12]. Ultrasound-guided endovascular stenting for the treatment of celiac artery dissection due to TAAD has been rarely reported in the literature [5]. Here, we present a case of a 39-year-old female with type A aortic dissection involving the celiac trunk. After open emergent ascending aortic arch replacement, she developed impending hepatic failure secondary to celiac artery occlusion within 48 hours postoperatively, requiring placement of a celiac artery stent. This article was previously presented as a meeting abstract at the 2024 Southern Medical Association Conference on February 17, 2024.

## Case presentation

**Figure 1:**
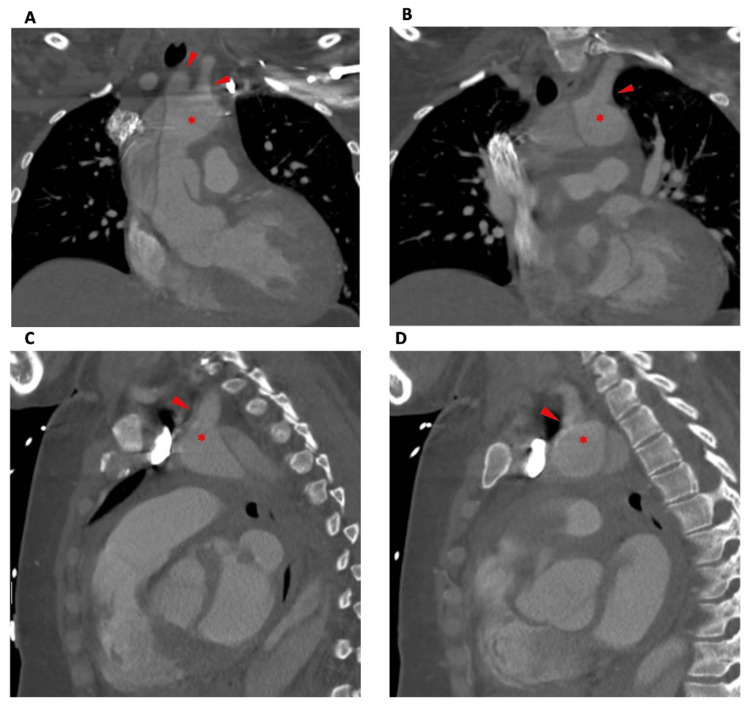
Type 1 aortic dissection with sparing of head vessels, seen on the CT scan on the day of admission Imaging shows the dissection beginning at the base of the ascending aorta and extending into the thoracic aortic arch, descending aorta, and the upper abdominal aorta. In the coronal series (A-B), the innominate and left common carotid arteries (A) and left subclavian (B) are shown. The dissection does not extend into these vessels. In the saggital series (C-D), the dissection can be clearly visualized distal to the left subclavian (C) and left common carotid artery (D); the dissection does not extend into these vessels (* indicates true lumen, red arrows indicate head vessels).

A 39-year-old female with a history of hypertension and prediabetes presented to the emergency department with severe acute chest pain radiating to the upper back. On exam, she was found to have a hypertensive emergency with equal radial pulses and no neurologic deficits. Computed tomography (CT) angiography of the chest showed a Stanford Type A, Debakey Type I aortic dissection sparing the head vessels (Figure 1).

**Figure 2:**
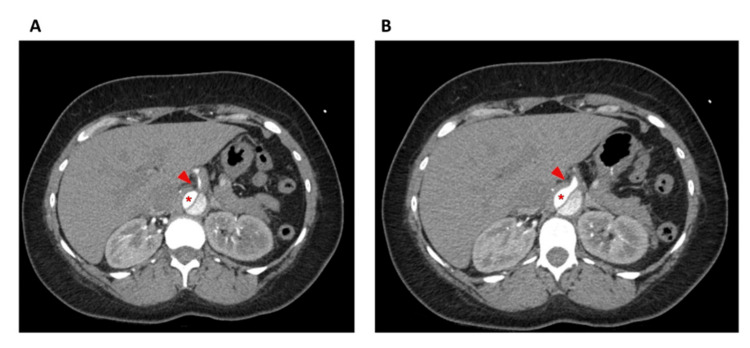
Computed tomography imaging demonstrated dissection notably extending into the celiac axis Red arrow shows celiac trunk; * indicates true lumen

The dissection began at the proximal portion of the ascending aorta distal to the aortic valve and extended into the thoracic aortic arch, descending aorta, and the upper abdominal aorta. Imaging demonstrated sparing of the arch vessels, with dissection notably extending into the celiac axis and superior mesenteric artery (SMA) (Figure 2).

She was transferred to our medical center and taken emergently to the operating room for open hemiarch repair from the sinotubular junction to the innominate artery. She was found to have a left ventricular ejection fraction of 55% with normal pulmonary artery pressures and sanguineous pericardial effusion without free myocardial wall rupture. After the pericardium was exposed, full heparinization was achieved and right axillary cannulation was established for CPB. Standard hypothermia was induced and an aortic cross clamp was applied. Myocardial preservation was maintained with retrograde blood cardioplegia and topical slush. A left ventricular vent was used.

The aorta was opened longitudinally and inspected. The dissection was found in the mid-ascending aorta. There was mild aortic insufficiency without disruption of the aortic valve. The patient was deeply cooled and circulation was reduced to minimal for cerebral perfusion. A direct distal graft anastomosis was initially performed. Since there was no involvement of the major branches, a simple tube graft was used to replace the aorta from the sinotubular junction to the innominate artery. Circulation was resumed and the proximal and graft-to-graft anastomoses were performed while rewarming.

The aortic cross clamp was removed, the patient resumed sinus rhythm, and atrial and ventricular pacing wires were placed. She was weaned from bypass without difficulty. In total, the patient was on CPB for 68 minutes and circulatory arrest for 23 minutes. Throughout the procedure, she remained in critical condition and was transfused with packed red blood cells, platelets, fresh frozen plasma, cryoprecipitate, Factor VII, estrogen, and desmopressin to maintain hemodynamic stability. Postoperatively, she remained intubated and was transferred to the intensive care unit (ICU) in critical condition with vasopressin and norepinephrine support.

**Figure 3:**
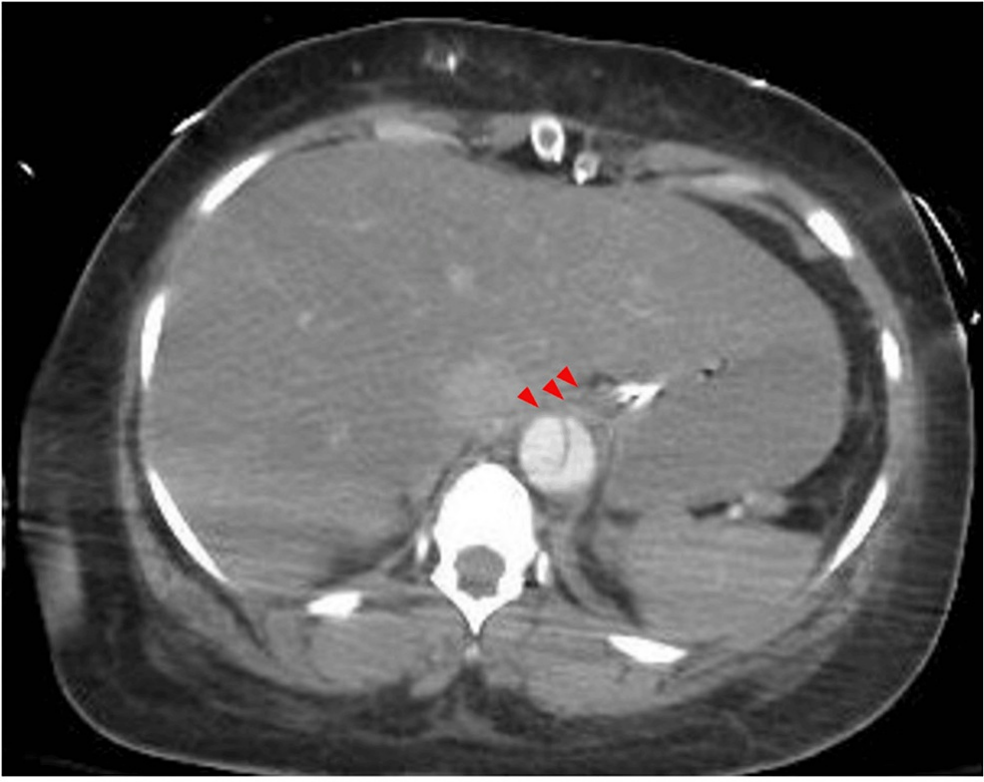
Computed tomography scan shows liver edema and ongoing dissection involving the celiac trunk Red arrows show celiac trunk

The day after her surgery, the patient developed acute kidney injury, leukocytosis, and an elevated lactate and international normalized ratio (INR). The CT scan of the abdomen and pelvis showed bowel edema, pelvic fluid, and ongoing dissection involving the celiac arterial trunk and SMA. Patent portal vein branches were visualized in the hilum of the liver, although the celiac artery appeared to have a central stenosis, presumably due to the dissection flap (Figure 3).

Due to concerns for bowel and hepatic ischemia, she was taken for emergent exploratory laparotomy approximately 30 hours after her initial surgery (day 3, Table 1). Intraoperatively, one liter of ascites was removed from the abdomen. The SMA had a palpable pulse, and the bowel appeared viable, but there was hepatic pallor. There was good flow in the portal vein, but a monophasic signal in the hepatic artery and absent Doppler signals within the liver parenchyma. These findings, combined with the knowledge that the dissection extended into the celiac trunk, as shown in Figure 3, were worrisome for a near-complete stenosis of the celiac arterial trunk. 

**Table 1:** Patient laboratory values demonstrating hepatic function over the course of hospital stay Postoperative day is relative to the date of aortic arch replacement (day 0). The patient was discharged on hospital day 21 with frequent follow-up visits. Data not available for the given date are indicated as N/A INR: International normalized ratio; AST: aspartate aminotransferase; ALT: alanine transaminase

Lab value	Day 1: Hemiarch Replacement	Day 2	Day 3: Stent Placement	Postoperative recovery						
				Day 4	Day 5	Day 6	Day 7	Day 10	Day 20	Day 50
											INR (ratio, no units)	1.1	0.9	2.9	2.9	2.4	1.9	1.5	1.3	1	Not Measured
Platelets (units/μL)	268000 units/μL	140000 units/μL	54000 units/μL	56000 units/μL	52000 units/μL	43000 units/μL	58000 units/μL	127000 units/μL	401000 units/μL	411000 units/μL
AST (U/L)	N/A	N/A	N/A	N/A	>5000 U/L	1892 U/L	609 U/L	58 U/L	50 U/L	36 U/L
ALT (U/L)	N/A	N/A	1766 U/L	936 U/L	230 U/L	128 U/L	88 U/L	37 U/L	48 U/L	28 U/L
Creatinine	1.06 mg/dL	2.18 mg/dL	6.35 mg/dL	5.52 mg/dL	3.28 mg/dL	3.19 mg/dL	3.6 mg/dL	5.4 mg/dL	4.69 mg/dL	0.92 mg/dL

The abdomen was closed and the patient was moved to the interventional suite next door within the same hospital where intravascular ultrasound was available. Endovascular repair was judged to be less invasive and safer than open repair for this patient who remained in critical condition. Left brachial artery access was obtained with ultrasound, and a narrow 6-French sheath was inserted.

**Figure 4:**
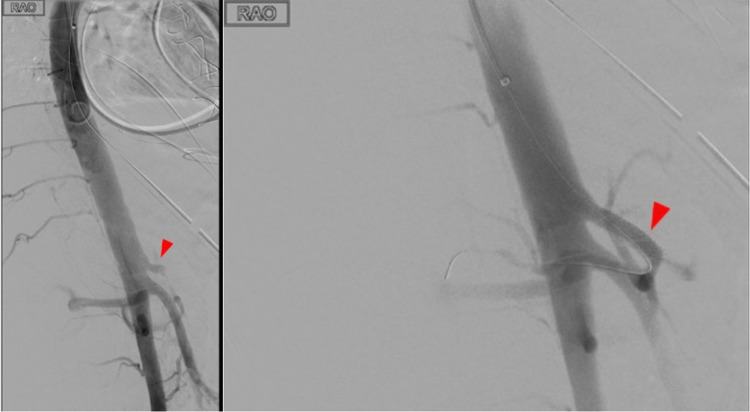
Angiogram showing celiac artery before stenting (left) and after stenting (right) The red arrow indicates the celiac trunk. The angiogram shows diminished blood flow through the mid to distal celiac arterial trunk. Flow was restored through the trunk to the common hepatic artery after the stent was deployed.

A pigtail catheter was floated across the aortic arch and positioned in the true lumen. Correct positioning was confirmed using a Pioneer Plus intravascular ultrasound device. An aortogram was obtained, which revealed the origin of the celiac artery with an approximately 99% stenosis (Figure 4). The renal arteries and SMA were noted to be patent. After heparinization, a 6-French, 70-cm angled sheath was placed. The celiac artery was accessed using a multipurpose angiographic catheter and Glide Advantage wire. The wire was advanced into the hepatic artery and subsequently the gastroduodenal artery, and selective angiography was used to confirm the positioning.

Next, the celiac artery was dilated using a 4-mm balloon, and a 6 x 27-mm balloon-mounted bare-metal stent was placed in the celiac artery extending into the common hepatic artery. Repeat angiography confirmed good flow through the stented artery. Figure 4 shows an angiogram before and after stenting of the celiac artery.

The sheath, catheters, and wires were removed, and external pressure was applied at the sheath insertion site for 15 minutes to achieve adequate hemostasis. The patient had a palpable left radial pulse at the end of the case. She was transferred back to the cardiovascular ICU in critical condition. Throughout the case, she received blood transfusions and underwent correction of acidosis.

**Figure 5:**
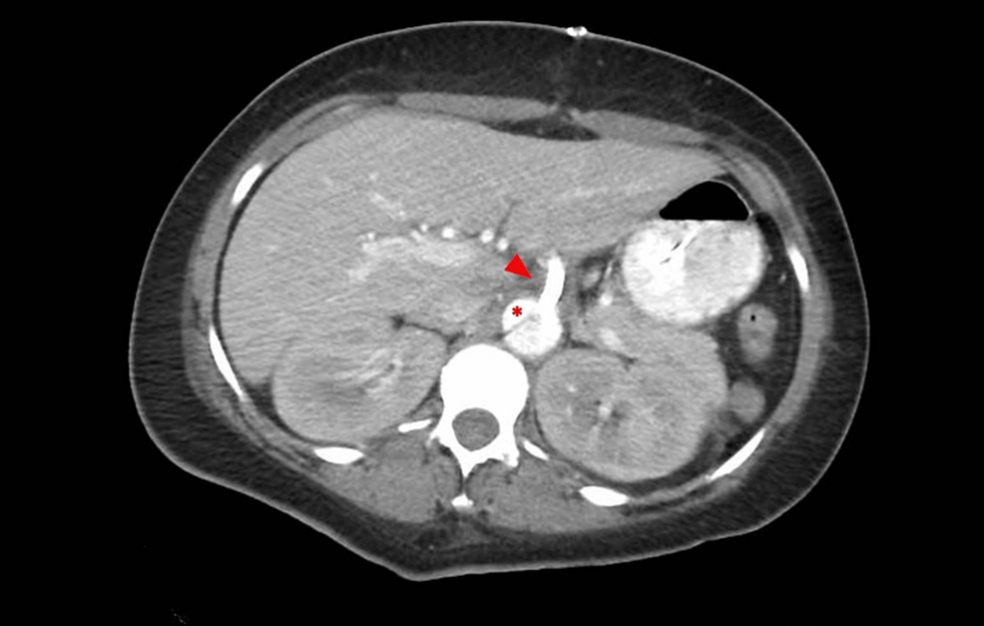
Postoperative computed tomography scan shows the patent celiac trunk stent. The red arrow indicates the celiac stent; * indicates true lumen

Postoperatively, the patient’s INR and hepatic function tests normalized (Table 1). Aspirin and clopidogrel were initiated on postoperative day 10 when the patient's platelet count passed 100,000 units/μL and INR stabilized at 1.3 to maintain patency of the celiac arterial stent. The patient has continued to take both daily during the 10 months of follow-up available at the time this article was submitted for publication. Postoperative CT imaging of the abdomen on day 13 showed that the celiac stent was patent (Figure 5).

Our patient’s overall clinical course was complicated by left hemispheric cerebral infarction, ventilatory dependency, and hemodialysis-dependent acute renal failure. Immediately after her operation, she was started on continuous renal replacement therapy until postoperative day 5, then every other day until day 20. Her renal function eventually recovered, and she was able to wean off of hemodialysis by day 41.

Per cerebral infarction, the patient was poorly responsive on day 2 after her initial hemiarch repair. CT scan on day 2 before both laparotomy and stenting showed left frontal hypodensity concerning for evolving stroke. Per neurological evaluation at the time, the most likely explanation was multifocal ischemic stroke secondary to embolism from aortic dissection. After her stent placement, she was able to open her eyes to voice and had spontaneous motor function in her left extremities but was unable to follow commands. An eventual cranial MRI on day 11 showed diffuse multifocal areas of acute ischemia involving bilateral hemispheres, including the cerebellum, most prominent in the left frontal lobe. On postoperative day 20, she was discharged to a neurological skilled nursing facility. The patient was seen in the vascular surgery clinic three months later and was found to have a successful recovery. She was independently ambulating, eating (her gastrostomy tube was removed), and speaking, with the exception of residual word-finding deficits.

## Discussion

Arterial branch occlusion secondary to aortic dissection, known as malperfusion syndrome, is a major cause of organ ischemia in the setting of type A or B aortic dissection. It affects approximately 30% of patients presenting with type A and 20% of those with a type B dissection [10,13,14,15]. Celiac artery dissection and stenosis can occur as a complication of aortic dissection, causing fatal end-organ damage if not corrected. In this patient with impending liver failure secondary to celiac arterial occlusion from an aortic dissection, celiac artery stenting allowed for re-perfusion of the liver and improved patient survival. Hepatic failure in the setting of aortic dissection can result from cardiac bypass, low cardiac output syndrome, and extended mechanical ventilation after TAAD [1,16,17]. One report details a patient with cardiac tamponade leading to severe right-sided heart failure and subsequent acute liver failure [16]. In a retrospective study involving 215 patients with acute TAAD, 60.9% developed postoperative hepatic dysfunction, associated with increased aortic cross-clamp time, respiratory dysfunction, and low cardiac output syndrome.

Low cardiac output syndrome after cardiac surgery leads to decreased organ perfusion, subsequent ischemia, and an increase in postoperative morbidity and mortality [1,17]. Postoperative respiratory dysfunction and prolonged mechanical ventilation after TAAD can also lead to liver hypoxia [1]. While reports involving TAAD are limited, celiac artery occlusion secondary to an extension of type B aortic dissection (TAAB) into the celiac artery has been reported in two case reports [5,13]. Kobayashi et al. described the use of two self-expandable stents (7/40 mm and 10/40 mm) in the celiac artery to successfully restore hepatic function in the setting of TAAB [5]. Nikolaos et al. noted the presence of celiac arterial dissection without end organ malperfusion after using the Arc of Buhler, a variant anastomosis between the superior mesenteric artery and the celiac arterial branches, rendering stent grafting unnecessary [13].

In addition to celiac artery involvement, aortic dissection may also result in SMA occlusion or thrombosis. One case report describes a TAAD dissection including the SMA and celiac arterial trunk, but perfusion to the celiac arterial trunk was restored following emergency interpositional ascending aortic grafting. Nonetheless, the SMA remained stenotic and the authors placed a 10x12 mm Genesis balloon expandable stent at the SMA orifice. This stent did not completely restore flow. Therefore, a longitudinal arteriotomy was performed for thrombectomy and a saphenous vein patch was used to close the arteriotomy [2]. 

In our case, while the preoperative CT scan seemed to show involvement of the SMA in the dissection, the SMA was noted to have a good pulse during the exploratory laparotomy, and the bowel appeared viable. Further, the intra-operative angiogram showed perfusion of the true lumen of the SMA and patency of the portal vein. Therefore, we do not believe that SMA occlusion was a major contributor to hepatic ischemia in this case. It is also possible that flow through the SMA and gastroduodenal collaterals contributed to hepatic recovery in this case. However, these collaterals were not adequate for hepatic perfusion before stent placement. Furthermore, hepatic function began to recover after insertion of the stent and re-opening of the hepatic artery.

Although our primary concern was the compromised hepatic function in our patient, we must also note that she also sustained an acute kidney injury requiring dialysis, contributing to the overall morbidity in this case. The main aortic dissection did not involve the renal arteries, which were noted to be patent on the visceral angiogram during stent placement. However, the dissection reduced the size of the true aortic lumen and thus blood flow to end organs. Further, our patient required CPB and minimal circulation during her initial hemiarch replacement procedure. Complications of CPB include platelet and coagulation cascade dysfunction, inflammation, and acute kidney injury [18]. Blood contact with the CPB machine, reperfusion injury, operative trauma, and release of native endotoxins can cause a systemic inflammatory response [18]. The inflammatory response and hypotension caused by CPB can lead to acute kidney injury, which may be treated with maintenance of high perfusion pressure and dialysis [18]. We believe that renal damage in this case was due to postsurgical acute kidney injury in the setting of CPB, reduced delivery of blood to the renal arteries due to aortic dissection, and minimal perfusion during hemiarch replacement.

Finally, our patient suffered from a postoperative stroke, which can occur in 1-10% of surgeries involving the aorta [19]. A multicenter study from 2014-2017 found that 13% of 8937 patients who underwent TAAD repair experienced postoperative stroke [20]. Embolus from aortic atherosclerosis and hypoperfusion are known causes of stroke after aortic surgery, both of which may have affected our patient [19]. Extension of an aortic dissection into head vessels can also cause stroke, but in our case, there was no extension of the dissection into the carotid arteries. It is possible that left brachial artery access contributed to anoxic brain injury in this case, but a CT scan done on day 2 before both laparotomy and stenting showed left frontal hypodensity concerning for evolving stroke. Per neurological evaluation at the time, the most likely explanation was multifocal ischemic stroke secondary to embolism from aortic dissection. Since stroke is a known complication of aortic surgery, patients and their caregivers should be made aware of these risks before surgery. Skilled nursing and neurological support enabled significant neurologic recovery for this patient.

## Conclusions

Patients with celiac artery occlusion and hepatic hypoperfusion after open aortic arch replacement surgery for TAAD require urgent reperfusion to prevent liver failure and mortality. Placement of a celiac artery stent within 48 hours of aortic arch replacement successfully corrected liver malperfusion for a 39-year-old woman with TAAD and a history of hypertension. Therefore, celiac artery stenting after aortic arch replacement can be a viable option for patients with TAAD with stenosis of the celiac artery.
